# Transmission of border disease virus from a persistently infected calf to seronegative heifers in early pregnancy

**DOI:** 10.1186/s12917-014-0275-7

**Published:** 2015-02-22

**Authors:** Ueli Braun, Monika Hilbe, Fredi Janett, Michael Hässig, Reto Zanoni, Sandra Frei, Matthias Schweizer

**Affiliations:** Department of Farm Animals, Vetsuisse-Faculty, University of Zurich, Winterthurerstrasse 260, CH-8057 Zurich, Switzerland; Institute of Veterinary Pathology, Vetsuisse-Faculty, University of Zurich, Winterthurerstrasse 260, CH-8057 Zurich, Switzerland; Institute of Veterinary Virology, Vetsuisse-Faculty, University of Bern, Länggass-Strasse 122, 3001 Bern, Switzerland; New Name: Institute of Virology and Immunology, Federal Food Safety and Veterinary Office, University of Bern, Länggass-Strasse 122, CH-3001 Bern, Switzerland

**Keywords:** Cattle, Border disease, Early pregnancy, Persistent infection, BVDV, Pestivirus

## Abstract

**Background:**

This study describes the transmission of border disease virus (BDV) from a persistently infected calf to seronegative heifers in early pregnancy, resulting in persistently infected fetuses. On day 50 of pregnancy (= day 0 of the infection phase), six heifers were co-housed in a free stall with a bull calf persistently infected with BDV (pi BVD) for 60 days. The heifers underwent daily clinical examination, and blood samples were collected regularly for detection of pestiviral RNA and anti-pestivirus antibodies. After day 60 (= day 110 of pregnancy), the heifers were slaughtered, and the fetuses and placentae underwent post-mortem and immunohistochemical examination and RT-PCR for viral RNA detection.

**Results:**

Three heifers had mild viraemia from day 8 to day 14, and by day 40 all heifers had pestivirus antibodies identified as anti-BDV antibodies in the serum neutralisation test. The placenta of the three viraemic heifers had histological evidence of inflammation, and fetal organs from these heifers were positive for pestivirus antigen by immunohistochemical examination and for BD viral RNA by RT-PCR and sequencing. Thus, co-housing of heifers in early pregnancy with a pi-BDV calf led to seroconversion in all heifers and persistent fetal infection in three.

**Conclusions:**

Considering that pi-BDV cattle can infect other cattle and lead to persistent infection of the fetus in pregnant cows, BDV should not be ignored in the context of the mandatory BVDV eradication and monitoring program. This strongly suggests that BDV should be taken into account in BVD eradication and control programs.

## Background

It has long been known that Border disease virus (BDV) is transmissible from sheep to cattle under experimental as well as natural conditions [1-9], but whether BDV transmission is possible from cattle to cattle has not been investigated. There was a recent report of a Galloway bull persistently infected with BDV, and because all heifers co-pastured with this bull were seropositive for BDV, it was suggested that the bull was responsible for BDV infection in this herd [9]. Not only the mode of transmission among cattle but also the clinical picture of bovine BDV infection remains unclear. The infected bull was examined for BDV because of retarded growth and poor fertility [9]. In addition, two heifers with Border disease were described with clinical signs resembling bovine virus diarrhoea virus (BVDV) infection and mucosal disease [3]. The goal of this study was to investigate the transmissibility of BDV from a calf persistently infected with BDV (pi-BDV calf) to seronegative heifers in early pregnancy and whether the fetuses of the heifers become persistently infected. A pi-BDV bull calf was housed with six seronegative heifers in early pregnancy for 60 days, after which time the heifers were slaughtered and the uteri and fetuses examined.

## Methods

### pi-BDV bull calf

The Braunvieh × Limousine pi-BDV bull calf originated from a BVDV-free herd of 24 cows, which were co-housed with 20 sheep in the same barn. With the exception of this calf, ear punch biopsy samples [10,11] of all cattle in the herd were negative for BVDV in an antigen ELISA (IDEXX BVDV Ag/Serum Plus Test, IDEXX Switzerland AG, Bern-Liebefeld, Switzerland) as part of the national BVDV eradication program [12]. Immunohistochemical evaluation revealed that the bull calf was positive for the pestivirus-specific antibody C16 but not for the BVDV-specific antibody Ca3/34-C42. RT-PCR evaluation (see below) of a blood sample was positive for pestiviral RNA and the calf was considered persistently viraemic. Radiographic findings of the bones of the extremities of the calf were described separately [13], animal no. 3). RT-PCR testing of blood samples of all other cattle of the herd were negative. Because the cattle were in contact with sheep, virus sequencing was initiated by the official veterinarian to characterise the virus, and BDV (BDSwiss, R8540/ 11_ch149) [14] was identified. The calf was acquired by our clinic when it was 41 days old. It was kept in quarantine until the age of 195 days, at which time it was moved along with the pregnant heifers to an isolation barn, where it remained until the end of the study.

### Heifers

Six open heifers of different breeds were acquired at the age of 382 to 748 days (means ± sd = 506 ± 126 days). Ear punch biopsy samples obtained from all the heifers had tested negative for pestivirus. The heifers were tested twice as seronegative by antibody ELISA as described below.

### Acclimation phase

The entire study period was divided into an acclimation and an infection phase. During the acclimation phase, the heifers were kept in quarantine without the pi-BDV calf. After estrus synchronisation, they were artificially inseminated using sperm from a BVDV-negative bull. Pregnancy was diagnosed ultrasonographically 30 days after insemination. Four of the heifers returned to estrus but conceived after the second insemination. The acclimation phase lasted until day 50 of pregnancy in each heifer.

### Infection phase

The infection phase was 60 days and lasted from day 50 (= day 0 of infection phase) to day 110 of pregnancy. During this phase the heifers were housed together with the pi-BDV calf in a separate free stall barn. Merging of the heifers with the calf was staggered because artificial insemination occurred on different days.

### Clinical examination

The heifers underwent thorough clinical examination before and at the end of the acclimation phase. In addition, the demeanour, behaviour, appetite and fecal consistency were monitored daily during acclimation. During the infection phase, the demeanour, appetite, circulatory, respiratory and digestive systems, mucous membranes and skin were assessed daily and the rectal temperature was recorded twice daily; the daily mean of the latter was calculated for each heifer. Ultrasonographic pregnancy examinations were carried out every ten days and the heifers were observed daily for signs of abortion.

### Blood sampling

EDTA blood for pestivirus antigen testing was collected on day 0 and then every other day until day 20 of the infection phase. Whole blood for pestivirus antibody testing was collected on day 0 and then every ten days until day 60 of the infection phase.

### Detection of viral RNA, pestivirus antibody and serum neutralisation test

RNA in the blood samples was isolated using a Bio-Robot-Universal-System (Qiagen AG, Hombrechtikon, Switzerland) and the QIAamp Virus BioRobot MDx Kit (Qiagen). RNA was detected according to the instructions of the Cador BVDV RT-PCR Kit (Qiagen) running as proposed for 45 cycles using a thermocycler ABI 7300 (Applied Biosystems, Rotkreuz, Switzerland). The primers and probes of this kit have a very high sensitivity for the detection of BVDV and BDV [15].

Following evaluation of the raw data, the amount of the viral RNA in the sample was expressed in Ct values; values of ≤30 were rated positive and values of >30 weakly positive. Blood samples were stored at 4°C, and weakly positive samples were re-tested after repeated RNA isolation.

An antibody ELISA developed at the Institute for Veterinary Virology, Vetsuisse Faculty, University of Bern [16], was used for pestivirus antibody detection in serum. After collection, the blood samples were stored at 4°C so that all samples from a heifer could be measured on the same day. The optical density (OD) was expressed as percentage of the OD of the standard serum; relative OD readings between 20% and 30% were considered indeterminate and those >30% were considered positive.

The serum neutralisation test (SNT) was used to identify the pestivirus species against which the antibody was directed [17]. The BDV type that was isolated from the bull calf (R8540/ 11_ch149) was used instead of the Moredun type. As cross reactions between BVDV and BDV are common based on their genetic relationship [18], only BDV titres at least four times higher than the BVDV titres were considered significantly higher [17].

### Macroscopic, histologic and immunohistochemical examinations

The uterus, placenta and ovaries of the heifers and fetal organs (large and small intestines, brain, skin, heart, liver, lung, spleen, umbilicus, kidneys, thyroid gland, thymus, forestomachs, tongue) were examined macroscopically and histologically. Tissues were fixed in formalin, embedded in paraffin, sectioned and stained with H&E and examined using light microscopy.

Samples of the thyroid gland, tongue, aural skin and other fetal organs, and of placentomes were collected for immunohistochemistry. Pieces of thyroid gland, tongue and skin were snap frozen in liquid nitrogen, cryosectioned and processed with the antibodies Ca3/34-C42 and C16 [11,19,20]. Samples of placentomes and other organs such as fetal brain were fixed in formalin and embedded in paraffin, and sections were processed with the antibodies C42 and 15c5 [20]. The antibody CA3/34-C42 (dilution 1:100; Labor Dr. Bommeli AG, Bern, Switzerland) and C42 (dilution 1:400; Prof. Moennig, Institute for Virology, Hannover, Germany) are specific for BVDV. The mixture of the antibodies Ca3/34-C42 binds to glycoprotein E2, and C42 binds to glycoprotein gp48 (E^rns^). The pestivirus-specific antibody C16 (dilution 1:100; Labor Dr. Bommeli AG) is directed against the nonstructural protein p125/80 (NS2-3/NS3), and the pestivirus-specific antibody 15C5 (dilution 1:10,000, E. Dubovi, New York State College of Veterinary Medicine, Cornell University, Ithaca, New York, USA) against the highly conserved glycoprotein gp48 (E^rns^).

### Virus detection in fetuses and placentae

Skin, thymus and small intestines from the fetuses and placentae were used for the detection of pestiviral RNA. Skin and thymus were disintegrated and homogenised mechanically using the Tissue Lyser (Qiagen) and small intestine and placenta enzymatically using the QIAamp Cador Pathogen Mini Kit according to manufacturer’s instructions. RNA isolation and sequencing of the 5’ terminal region (5’-UTR) of the pestiviral genome was done as described [21]. The same RNA was also used to detect the viral genome at the 5’-UTR using RT-PCR (Cador BVDV RT-PCR Kit, Qiagen) and traditional RT-PCR [20].

### Statistical analysis

Data were recorded in Office Excel 2007 (Microsoft Inc.). Descriptive statistics were used to describe continuous data (IBM SPSS Statistics 20, IBM Switzerland AG, Zürich) and normality was tested using the Wilk-Shapiro test. Means ± standard deviations were calculated for normally distributed data and medians, minimum and maximum values for data with non-normal distribution. The program STATA 12 (StataCorp., 2011, Stata Statistical Software, Texas, USA) was used to analyse OD values (antibody titres). A *t*-test, a general linear model and ANOVA were carried out to analyse differences in OD values as a function of day of the infection phase and presence of a pi fetus. The underlying Stata model for the *t*-test was < by varx, sort: ttest vary, by (varx2)>, whereby varx = day variable, vary = OD value, varx2 = independent variable, for the general linear model it was < xtmixed vary varx2##varx || varx:> and for the ANOVA it was < vary varx3 c.varx2, repeated (varx) bse (varx4)>, whereby varx3 = heifer and varx4 = time point. A P-value ≤0.05 was considered significant.

### Approval of the study by an ethical committee

The study was approved by an ethical committee of the canton of Zurich, Switzerland.

## Results

### Clinical findings

The pi-BDV calf was clinically healthy and afebrile but grew very little during the study period. Radiographic studies revealed a mild increase in radiopacity of the diaphyses, which extended to the metaphyses, and mild osteopetrosis [13], animal no. 3. Except for week 4 of the infection phase, the six heifers were clinically healthy during the entire period; four heifers (nos. 1 to 4) had enzootic bronchopneumonia in week 4, which was most likely introduced by heifer 4. Heifers 5 and 6 were not in the infection phase at that time and were not affected. The details of the daily clinical examinations have been reported [22]. The rectal temperature varied from 37.9 to 40.1°C (median =38.5°C) during the infection phase. In three heifers, the rectal temperature exceeded 39.0°C on days 14, 29, 30 (heifer 1), day 22 (heifer 2) and days 6, 7, 10 and 60 (heifer 4). Heifer 4 was moved in with the first three heifers one week before the first increases in rectal temperature were noted; with two exceptions (heifer 1, day 14; heifer 4, day 60) all temperature spikes occurred in the fourth week of the infection phase and were attributed to enzootic bronchopneumonia. There were no cardiovascular system abnormalities, and the respiratory rate ranged from 20 to 52 breaths per minute (median =28 bpm). Four heifers had increased bronchial sounds on days 31 and 33 (heifer 1), 22 to 32 (heifer 2), 25 to 30 (heifer 3) and 7 to 17 (heifer 4). The heifers also had wheezes on days 22 to 32 (heifer 2) and day 27 (heifer 3). Abnormal lung sounds were accompanied by coughing, which persisted to day 60 in heifer 4. Abnormalities of the skin and mucous membranes such as erythema and erosions were not seen. Ruminal motility, intestinal sounds and feces were normal and no abnormal vaginal discharge or signs of abortion were noticed. Pregnancy was confirmed and fetal heart beat observed ultrasonographically at each examination.

### Virus detection in blood

Three heifers had weakly positive Ct values ranging from 37.8 to 42.5, indicative of pestiviral RNA and thus viraemia on day 10 (heifer 3), day 8 (heifer 4) and days 8 to 14 (heifer 5; Table 1). Re-examination of the weakly positive blood samples yielded positive results for heifers 4 and 5 on days 8 and 10, respectively, and negative results for the remaining samples.

**Table 1:** **Detection of viral RNA (Ct values) in EDTA blood from 3 heifers in early pregnancy on days 8 to 14 of the infection phase**

		**Ct value**		
**DayTag**	**Assay**	**Heifer 3**	**Heifer 4**	**Heifer 5**
8	Primary	Negative	37.8	40.6
	Follow-up	Negative	39.7	Negative
10	Primary	40.7	Negative	39.6
	Follow-up	Negative	Negative	41.7
12	Primary	Negative	Negative	42.5
	Follow-up	Negative	Negative	Negative
14	Primary	Negative	Negative	40.8
	Follow-up	Negative	Negative	Negative

Ct values of >30 were considered weakly positive.

Viral RNA was not isolated from heifers 1, 2 and 6.

Viral RNA was not isolated from heifers 3, 4 and 5 on days 0 to 6 and 16 to 20.

### Antibody detection in blood (ELISA)

**Figure 1:**
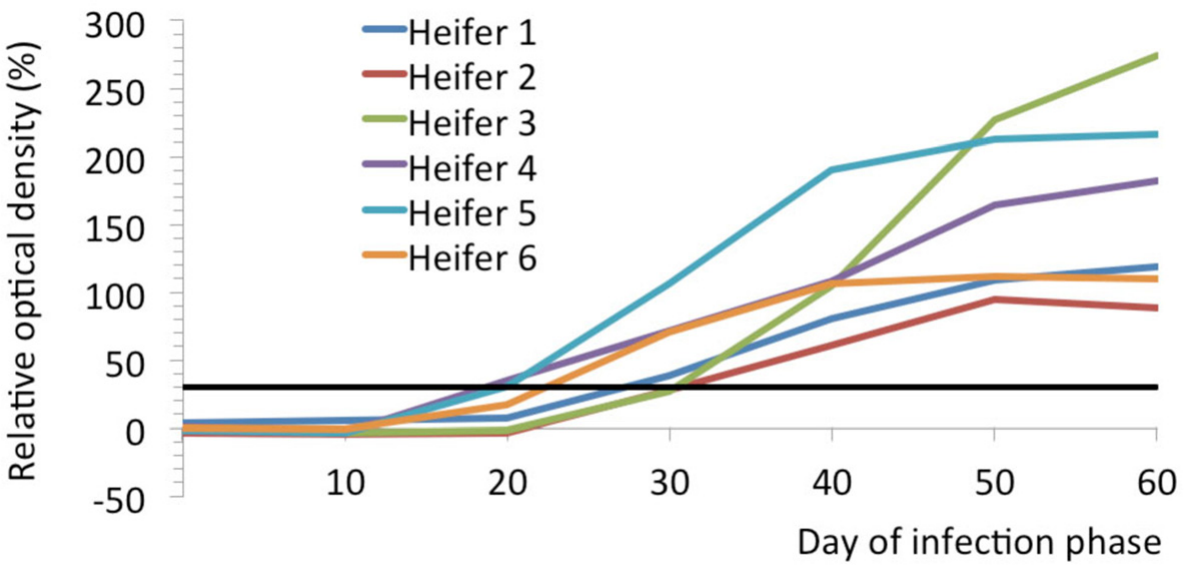
**Relative optical density from day 0 to day 60 of the infection phase.** Relative optical density (OD) expressed as percentage of the optical density of a standard serum in six heifers in early pregnancy from day 0 to day 60 of the infection phase. Relative OD values >30% (black line) are defined as positive.

All heifers were seronegative during the first ten days of the infection phase (Figure 1). In heifers 4, 5 and 6, the relative OD increased gradually between days 11 and 19, and in heifers 1, 2 and 3, it increased after day 20. On day 20, heifers 4 and 5 had an OD of 35% and 30%, respectively, which indicated seroconversion. Heifers 1 and 6 seroconverted by day 30 (OD 39%, 71%) and heifers 2 and 3 by day 40 (OD 61%, 104%). The relative OD increased in all heifers until day 60, at which time maximum relative OD values were measured in heifers 3 (274%), 4 (182%) and 5 (216%, Table 2). Heifers 1, 2 and 6 had values of 119%, 89% and 110%.

**Table 2:** **Relative OD values, SNT titres and quotients of BDV and BVDV SNT titres of 6 heifers in early pregnancy on day 60 of the infection phase**

**Heifer**	**OD value (%)**	**SNT BDV**	**SNT BVDV**	**Quotient of BDV and BVDV titres**
1	119	152	≤ 4	≥ 38
2	89	215	≤ 4	≥ 53
3	274	304	8	38
4	182	431	27	16
5	216	> 512	16	> 32
6	110	> 512	8	> 64

### Serum neutralisation test

Serum samples collected on day 60 were positive for BDV in all heifers. Titres of specific neutralising BDV antibodies were high and varied from 152 to >512 (Table 2). In two heifers (1 and 2), the SNT was negative for BVDV and the remaining four heifers had very low titres between 8 and 27. The quotient of BDV and BVDV antibody titres was greater than 4 in all heifers and indicated antibody production in response to BDV infection.

### Examination of uterus, placenta and fetus

**Figure 2:**
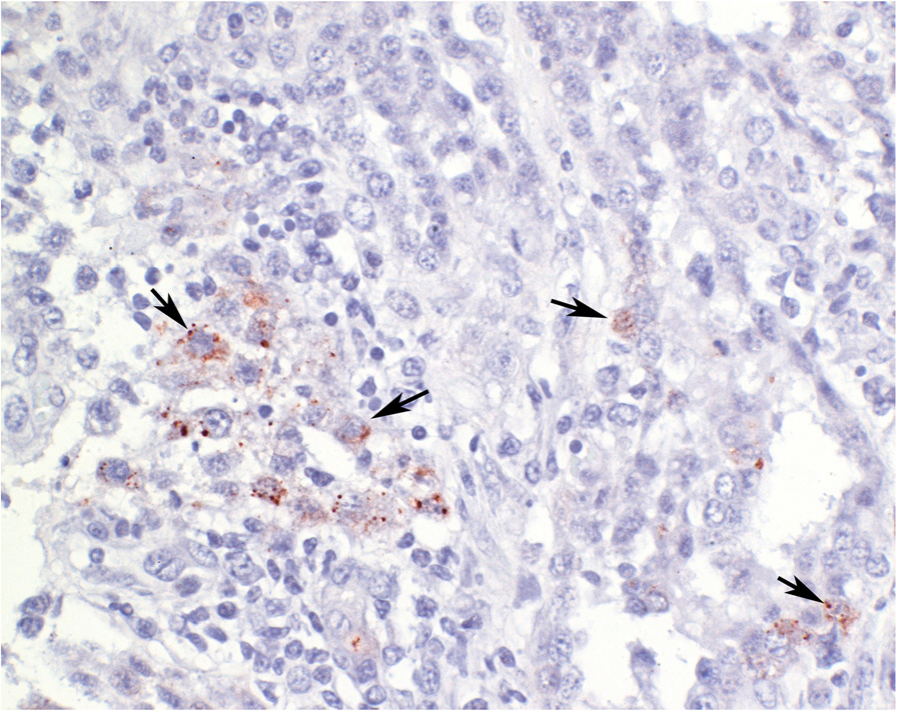
**Immunohistochemical staining of a placentome.** Immunohistochemical staining of a placentome from heifer 5 (Dako EnVision staining system, antibody 15C5, hemalaun counterstain). The cytoplasm of pestivirus-positive fetal cells is stained red (arrows).

All uteri, placentae and fetuses were macroscopically normal, and fetal organs were also histologically normal. The placentomes of heifers 3, 4 and 5 had multifocal to diffuse plasmacytic and lymphocytic infiltration and pronounced fibrosis, and those of heifers 4 and 5 also had moderate necrotic changes (Table 3). In heifers 3, 4 and 5, fetal organs and placentomes were positive for the pestivirus-specific antibodies C16 and 15C5 (Table 3) and negative for the BVDV-specific antibodies C42, CA3 and CA34. Notably, pestivirus-specific immunohistochemical staining was predominantly located in fetal cells of the placentomes and was scarce in maternal cells (Figure 2). Placentomes of heifers 1, 2 and 6 were negative when tested with pestivirus- and BVDV-specific antibodies.

**Table 3:** **Histological, immunohistochemical (IHC) and virologic (RT-PCR) findings of the placentomes from 6 heifers in early pregnancy and fetal organs**

		**Finding**			
**Heifer**		**Histology**	**IHC**	**RT-PCR**	**Fetal infectious status**
1	Heifer	Negative	Negative	Negative	NA
	Fetus	Negative	Negative	One organ weakly positive	Not infected
2	Heifer	Negative	Negative	Positive	NA
	Fetus	Negative	Negative	Partially positive	Transiently infected
3	Heifer	I, F	Positive	Positive	NA
	Fetus	Negative	Positive	Positive	Persistently infected
4	Heifer	I, F, N	Positive	Positive	NA
	Fetus	Negative	Positive	Positive	Persistently infected
5	Heifer	I, F, N	Positive	Positive	NA
	Fetus	Negative	Positive	Positive	Persistently infected
6	Heifer	Negative	Negative	Partially positive	NA
	Fetus	Negative	Negative	Partially positive	Transiently infected

IHC immunohistochemical staining with antibody C16/15C5.

I inflammation, F fibrosis, N necrosis.

NA Not applicable.

Real-time and traditional PCR detected pestiviral RNA sequences in fetal organs and placentomes of heifers 3, 4 and 5 (Table 3). In heifers 3 and 5, the sequence of the 5’ terminal region was identical to the sequence isolated from the pi-BDV calf. In all organs of heifer 4, there was a base-pair substitution of thymine for adenine at position 94. Pestivirus detection was limited to the small intestines of the fetus in heifer 1 but because the amount of RNA was too small for sequencing, contamination of the sample could not be ruled out. The placentomes of heifer 2 and skin and small intestine of her fetus yielded pestiviral RT-PCR products with sequences that were identical to the viral sequence of the pi-BDV calf, but the RT-PCR results of the fetal thyroid gland were not conclusive. The placentomes and small intestine of the fetus of heifer 6 also yielded several products corresponding to pestiviral RNA. The sequence of pestiviral PCR products from the placenta were identical to those from the pi-BDV calf and the products of the small intestine contained the same mutation as that described for the products from heifer 4.

Based on the unequivocally positive results of the immunohistochemical and virologic examination of all fetal organs, the fetuses from heifers 3, 4 and 5 were diagnosed as persistently infected with BDV, though we cannot predict whether these fetuses would have developed to full term and be born alive as persistently infected calves. The fetus of heifer 1 was classified as not infected with BDV because immunohistochemistry and RT-PCR did not allow reliable detection of the virus. Likewise, the fetuses of heifers 2 and 6 were not considered persistently infected although pestivirus was detected in some fetal organs; Real-time PCR was negative or the Ct values were greater than 30, the bands in the agarose gel following traditional PCR amplification were faint or absent and immunohistochemical analysis, which in most cases identifies only PI animals [20], was negative. This led to a tentative diagnosis of transient BDV infection.

### Relationship between seroconversion and day of infection and presence of a persistently infected fetus

**Figure 3:**
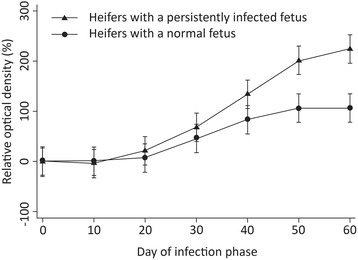
**Mean relative OD of the pestivirus antibody ELISA for heifers with a persistently infected fetus and heifers with a normal fetus.** Mean relative OD of the pestivirus antibody ELISA and 95% confidence intervals for 3 heifers with a persistently infected fetus (▲) and 3 heifers with a normal fetus (●). * P ≤0.05.

Analysis of the mean relative OD during the 60 days of the infection period (6 heifers × 7 measurements) with the generalised linear model revealed a significant effect of day of infection phase and status of fetal infection (infected or not infected) on relative OD. The *t*-test revealed significant differences between heifers with a persistently infected fetus and those without with respect to mean relative OD values on days 50 and 60 (Figure 3). Based on the generalised linear model, heifers with a persistently infected fetus had significantly higher mean relative OD than heifers without a persistently infected fetus from as early as day 40. The 90%-confidence intervals of the relative OD overlapped slightly on day 40 but clearly diverged on days 50 and 60. There was no temporal relationship between day of seroconversion and respective clinical findings.

## Discussion

Pestiviral RNA was isolated from three heifers (3, 4, 5) on several occasions between days 8 and 14. Re-examination of the samples confirmed infection in two heifers (4, 5) but not in the third, which had only been weakly positive once on day 10. These findings support pestiviral viraemia, albeit at a low level. The time frame of the viraemia was similar to that observed in calves (8 to 21 days) [23,24], sheep (2 to 21 days) [25], and lambs (4 to 9 days) [26] infected with BDV. The observation that pestiviral RNA was only isolated from heifers with a persistently infected fetus and that, at the same time, display the highest antibody titres, supports the presence of true viraemia. This is therefore the first report of BDV viraemia in cattle after contact with a persistently infected animal of the same species. Calves and heifers co-housed with sheep persistently infected with BDV seroconverted but did not become viraemic [5,7,24,27]. On the other hand, viral RNA could be isolated from blood of calves orally infected with BDV [13,24].

Two heifers seroconverted as early as day 20, and the remaining heifers seroconverted by day 40. This variability may have been the result of differences in susceptibility to BDV, viral dose and time of exposure to the virus, but was similar to the variability in pregnant heifers that seroconverted 23 to 38 days after first contact with sheep persistently infected with BDV [27]. Heifers with a persistently infected fetus had significantly higher titres from day 40 than heifers without an infected fetus, and there was a significant effect of duration of viraemia in the heifers and infectious status of the fetus on mean relative OD over the entire study period. The effect of a persistently infected fetus on antibody titre has not been described for BDV infection but has been known for BVDV infection; cows carrying a fetus persistently infected with BVDV had antibody titres at day 180 of pregnancy that were on average ten times higher than in cows with a non-infected fetus [28]. Similarly, the comparison of cows with and without a fetus persistently infected with BVDV showed that OD values were significantly related to duration of pregnancy, time of sampling and fetal infectious status [29,30]. Antibody titres rose much faster when the fetus was persistently infected, and after day 135 of pregnancy, OD differed significantly between the two groups of cows [30].

Serum neutralisation testing indicated that all heifers had high titres of specific antibodies against BDV at the end of the infection phase. Four heifers also had low antibody titres against BVDV, which was thought to be due to slight cross-neutralisation within the pestivirus genus. Nonetheless, titres against BDV were 16 to 64 times higher than the titres against BVDV, indicating that the heifers were infected with BDV as a BDV titre at least four times higher than a BVDV titre is considered to be specific [31].

All uteri, placentae and fetuses were macroscopically normal and the fetal organs were also histologically normal. There was histological evidence of inflammation in the placentomes of the three heifers carrying an infected fetus, similar to lesions seen in BDV-infected pregnant sheep [32] and cows [27,33] and in BVDV-infected pregnant cows [32]. It can be assumed that the fetal infection originated from the placenta.

Immunohistochemical examination of tongue, skin, thyroid gland and placenta is an established and sensitive method for the detection of persistent fetal pestivirus infection [19]. Three of the six fetuses and placentae were positive for the pestivirus-specific antibodies C16 and 15C5 but negative for the BVDV-specific antibodies C42, CA3 and CA34, and it is therefore most likely that these three fetuses were persistently infected with BDV. Interestingly, staining was all but limited to the fetal part of the placentome. Heifers 3, 4 and 5 had high titres against BDV from days 20 to 40, which could have neutralised the viral antigen in the maternal circulation. Because the passage of molecules across the epitheliochorial bovine placental barrier is highly selective [34], BDV antibodies may have been prevented from entering the fetal circulation, thus allowing infection of the fetus to occur.

Reverse transcriptase PCR allowed confirmation of BDV infection of the fetuses from heifers 3, 4 and 5. The RNA sequence isolated from heifers 3 and 5 was identical to the sequence isolated from the pi-BDV calf. The base-pair substitution of thymine for adenine at position 94 might be based on a RNA-virus specific characteristic whereby viruses produced within an infected cell are not always identical but may vary because of mutation and recombination within the viral genome [35]. Similar changes in amino acid sequences over time and the generation of variant viruses were observed in two pi-BVDV calves [36]. Isolation of pestiviral RNA from fetal organs and placentomes of heifers 2 and 6 could possibly have been based on true infection or on contamination of the sample. Assuming that persistent BDV and BVDV infections have similar underlying mechanisms, immunohistochemical examination of fetuses persistently infected with BDV should have yielded positive results for viral antigen. In bovine fetuses persistently infected with BVDV, immunohistochemical staining of fetal organs was as sensitive as virus isolation [19,37] and RT-PCR [20]. Therefore, we suspect that the fetuses of heifers 2 and 6 were transiently infected, which is supported by the fact that the isolation of RNA from organs of calves transiently infected with BVDV has been reported [20,38]. Isolation of pestiviral RNA from the fetus and placentome of heifer 1 was not conclusive but based on the seroconversion similar to heifers 2 and 6, it has to be assumed that heifer 1 became infected after contact with the pi-BDV calf.

## Conclusion

Considering that pi-BDV cattle can infect other cattle and lead to persistent infection of the fetus in pregnant cows, BDV should not be ignored in the context of the mandatory BVDV eradication and monitoring programs such as the one instituted in Switzeland. In Switzerland and other countries, sheep and cattle are commonly kept in the same barn or co-pastured on alpine summer pastures, and it is therefore likely that BDV is transmitted from sheep to cattle and subsequently from the infected cattle to other cattle. For this reason, testing for BDV should be included in BVD eradication and control programs. It is doubtful whether pestiviruses in cattle can be eradicated without differentiating BVDV and BDV infection and the inclusion of other susceptible species such as sheep in the Swiss control program. It is therefore possible that pestivirus in cattle can not be eradicated without differentiating BVDV and BDV infection and the inclusion of other susceptible species such as sheep in the Swiss control program.
